# Clinical characteristics and factors associated with in-hospital mortality in patients with *Stenotrophomonas maltophilia* infection: a single-center retrospective cohort study

**DOI:** 10.3389/fmed.2026.1902452

**Published:** 2026-08-21

**Authors:** Hanli Wang, Min Xing, Haoyu Ji, Yusheng Cheng, Shuhui Hu, Guofeng Wang, Susheng Zhou, Yonghui Han, Bohan Zhang, Lei Zha, Qinghai You

**Affiliations:** 1 Department of Respiratory and Critical Care Medicine, The First Affiliated Hospital of Anhui Medical University, Hefei, Anhui, China; 2 Department of Respiratory Medicine, The First Affiliated Hospital of Wannan Medical University, Wuhu, Anhui, China; 3 Department of Respiratory Medicine, The First Affiliated Hospital of Anhui Medical University North District, Hefei, Anhui, China; 4 Department of Interventional Pulmonology, Anhui Chest Hospital, Hefei, Anhui, China; 5 Anhui Medical University, Hefei, Anhui, China

**Keywords:** antimicrobial susceptibility, carbapenem exposure, Charlson comorbidity index, empirical antimicrobial therapy, in-hospital mortality, *Stenotrophomonas maltophilia* infection

## Abstract

**Background:**

*Stenotrophomonas maltophilia* is an intrinsically multidrug-resistant opportunistic pathogen that causes clinically significant infections, particularly in immunocompromised and critically ill patients. The independent contributions of prior antimicrobial exposure and early active therapy to mortality remain uncertain. This study evaluated clinical characteristics, antimicrobial exposure patterns, empirical treatment appropriateness, and mortality-associated factors in patients with clinically confirmed *S. maltophilia* infection.

**Methods:**

This single-center retrospective cohort study included 458 hospitalized patients with confirmed *S. maltophilia* infection at a tertiary hospital between 1st June 2023 to 31st July 2025. Demographic characteristics, comorbidities, infection-related variables, prior antimicrobial exposure, empirical treatment regimens, laboratory indicators, antimicrobial susceptibility results, and in-hospital outcomes were extracted from electronic medical records. Univariable and multivariable logistic regression analyses were performed to identify factors associated with all-cause in-hospital mortality, with particular attention to prior carbapenem exposure and the appropriateness of initial empirical antimicrobial therapy.

**Results:**

Overall in-hospital mortality was 11.8% (54/458). Compared with survivors, non-survivors were older, had a higher Charlson Comorbidity Index (CCI), and more frequently presented with severe clinical features, including septic shock, mechanical ventilation, central venous catheterization, and elevated inflammatory markers. In the multivariable model, older age and higher CCI remained independently associated with mortality, whereas mechanical ventilation showed a strong but statistically non-significant association. Prior carbapenem exposure was common, but mortality was comparable between carbapenem-exposed and unexposed patients. The appropriateness of initial empirical antimicrobial therapy was not independently associated with survival after adjustment for baseline and severity-related factors. Antimicrobial susceptibility testing showed high *in vitro* activity for minocycline, cefoperazone/sulbactam, doxycycline, and tigecycline.

**Conclusions:**

In this cohort of patients with *S. maltophilia* infection, mortality was associated primarily with host vulnerability and illness severity rather than prior carbapenem exposure or empirical treatment appropriateness alone. Prior carbapenem use may reflect underlying disease complexity rather than act as an independent driver of mortality. These findings suggest that clinical management should emphasize early risk stratification, careful assessment of comorbidity burden, timely supportive care, and antimicrobial selection guided by local susceptibility patterns.

## 1 Introduction

*Stenotrophomonas maltophilia* is an opportunistic, non-fermenting Gram-negative bacillus that is increasingly recognized as a clinically important healthcare-associated pathogen, particularly in settings characterized by prolonged hospitalization, broad-spectrum antimicrobial exposure, invasive devices, and intensive care utilization (1, 2). It is most often encountered in patients with impaired host defenses, hematologic malignancies, chronic organ dysfunction, invasive procedures, prolonged hospitalization, or intensive care unit (ICU) exposure (3, 4). The organism is environmentally versatile and capable of adapting to healthcare and host-associated niches, which contributes to its persistence in hospital settings (5, 6). Although *S. maltophilia* has historically been considered to have relatively low intrinsic virulence, its clinical importance is substantial in vulnerable patients, in whom it can cause pneumonia, bloodstream infection, urinary tract infection, skin and soft tissue infection, intra-abdominal infection, and other severe infectious syndromes (7).

The clinical management of *S. maltophilia* infection is complicated by microbial resistance, biofilm formation, virulence-associated capabilities, and host-related factors. Its intrinsic resistance to multiple antimicrobial classes is mediated by chromosomally encoded beta-lactamases, multidrug efflux pumps, reduced outer-membrane permeability, and other adaptive resistance mechanisms (8–10). The organism can also adhere to epithelial and abiotic surfaces and form biofilms, which may facilitate persistence in respiratory and device-associated niches (11, 12). Genomic studies further suggest that hospital-associated *S. maltophilia* populations are diverse and may spread nosocomially under appropriate ecological pressure (13). These features restrict empirical treatment options and may delay the initiation of active therapy, especially in critically ill patients who have already received broad-spectrum antibiotics. However, mortality after *S. maltophilia* infection is unlikely to be determined by antimicrobial susceptibility alone. Advanced age, comorbidity burden, immune dysfunction, organ failure, mechanical ventilation, and septic shock can substantially influence prognosis, particularly when the organism emerges in patients with limited physiological reserve.

Previous studies have described the epidemiology, antimicrobial susceptibility patterns, and clinical risk factors associated with *S. maltophilia* infection, but the relative contribution of treatment-related factors to mortality remains uncertain. Prior carbapenem exposure is clinically relevant because it may reflect antimicrobial selection pressure and disruption of competing flora. At the same time, carbapenem exposure may also serve as a marker of severe underlying illness rather than as an independent determinant of death. Similarly, appropriate initial empirical therapy is clinically important, but its measurable effect on survival may be attenuated in retrospective cohorts when baseline comorbidities and acute disease severity dominate the clinical course.

Evidence from Chinese real-world hospital settings remains limited, particularly from studies that jointly evaluate host factors, disease severity, prior antimicrobial exposure, empirical treatment appropriateness, and antimicrobial susceptibility profiles within the same cohort. Therefore, this single-center retrospective cohort study included 458 patients with confirmed *S. maltophilia* infection between 1st June 2023 and 31st July 2025. The aims were to characterize the clinical and microbiological features of these patients, identify factors associated with all-cause in-hospital mortality, and evaluate whether prior carbapenem exposure and appropriate empirical antimicrobial therapy were independently associated with survival after adjustment for host vulnerability and illness severity.

## 2 Materials and methods

### 2.1 Study design, setting, and case identification

This single-center retrospective cohort study was conducted at The First Affiliated Hospital of Wannan Medical University, a tertiary hospital in Wuhu, Anhui Province, China. Consecutive hospitalized patients with at least one clinical specimen positive for *Stenotrophomonas maltophilia* between 1 January 2023 and 31 July 2025 were screened through the hospital microbiology laboratory information system and electronic medical records.

**Figure 1:**
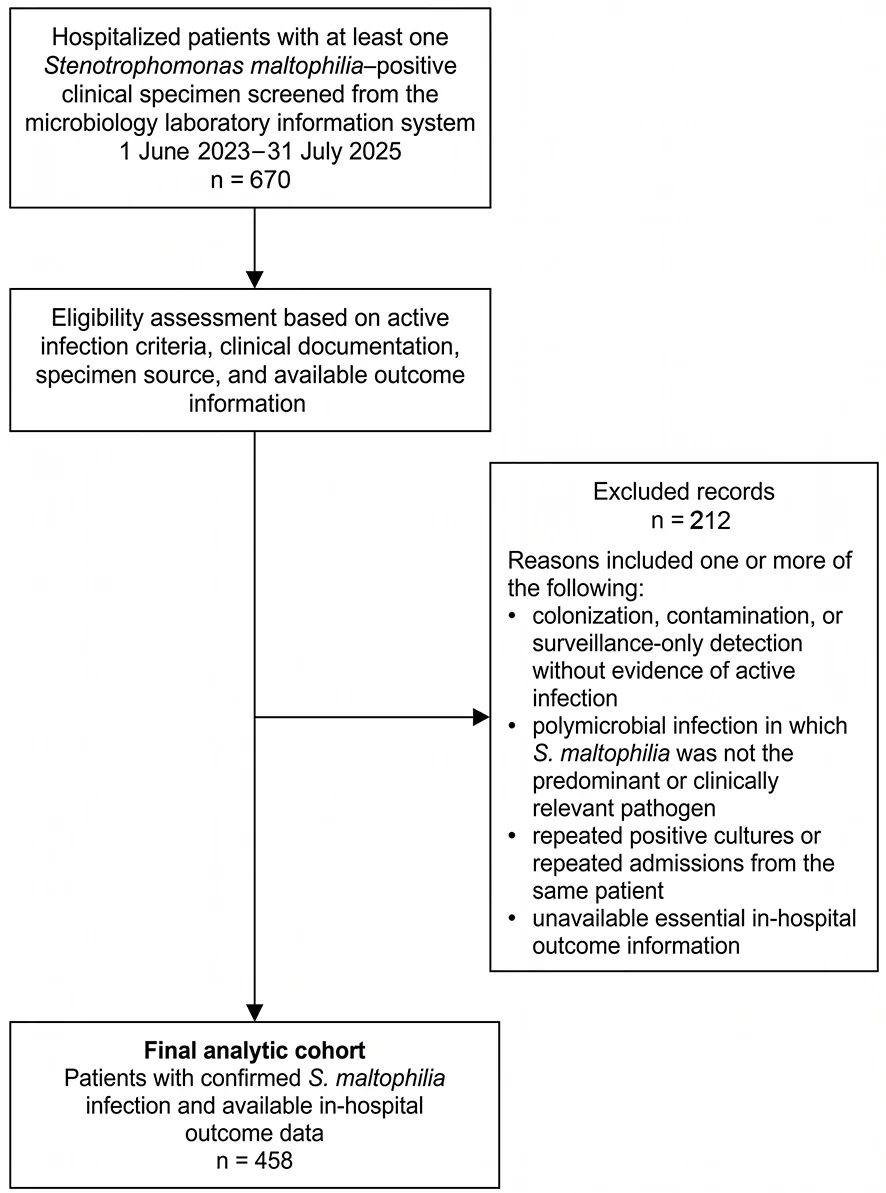
Flow diagram of patient screening and cohort inclusion.

The index date was defined as the specimen collection date of the first eligible *S. maltophilia*-positive culture during the index hospitalization. The culture report date was not used as the index date because baseline clinical status, prior antimicrobial exposure, empirical treatment classification, laboratory indicators, and outcome intervals were defined relative to specimen collection. When more than one positive culture was recorded during the same hospitalization, only the first eligible isolate was analyzed. When a patient had repeated hospitalizations during the study period, only the first hospitalization meeting the eligibility criteria was included. The screening, exclusion, and final cohort inclusion process is shown in Figure 1.

Consecutive hospitalized patients with at least one *S. maltophilia*-positive clinical specimen between 1st June 2023 and 31st July 2025 were screened. Patients were excluded if the isolate represented colonization, contamination, or surveillance-only detection without evidence of active infection; if *S. maltophilia* was not considered the predominant or clinically relevant pathogen in a polymicrobial infection; if repeated episodes occurred in the same patient; or if essential outcome information was unavailable. The remaining patients constituted the final retrospective cohort for analysis.

### 2.2 Eligibility criteria

Patients were eligible for inclusion if they met all of the following criteria: (1) inpatient admission during the study period; (2) isolation of *S. maltophilia* from at least one clinical specimen; (3) clinical evidence supporting active infection rather than colonization; and (4) available in-hospital outcome information. Active infection required microbiological confirmation plus compatible clinical evidence, including relevant symptoms or signs, inflammatory or organ-function abnormalities, imaging findings when applicable, and physician-documented infection in the medical record. Antimicrobial initiation or continuation was considered supportive clinical context but was not used as the sole criterion for defining active infection, and infection classification was completed before assessment of empirical therapy appropriateness.

Patients were excluded if the positive culture was judged to represent colonization, contamination, or surveillance-only detection without evidence of active infection. Patients were also excluded if *S. maltophilia* was isolated in a polymicrobial infection but was not considered the predominant or clinically relevant pathogen; this determination was standardized using objective microbiological criteria, requiring moderate to heavy semi-quantitative growth on primary culture plates and congruent Gram stain findings demonstrating abundant Gram-negative bacilli alongside polymorphonuclear leukocytes. Patients were additionally excluded if essential outcome information was unavailable. Infection classification was based on established healthcare-associated infection criteria and site-specific clinical assessment (14). The study protocol was approved by the Ethics Committee of The First Affiliated Hospital of Wannan Medical University (approval No. 2024-83).

### 2.3 Infection site and distinction from colonization

The primary infection site was determined according to specimen type, clinical diagnosis, physician documentation, and the dominant clinical syndrome at the index date. Infection sites were categorized as pulmonary infection, bloodstream infection, urinary tract infection, skin and soft tissue infection, intra-abdominal infection, or other documented sites.

If more than one potential infection site was documented, the primary site was assigned according to the main clinical diagnosis recorded around the index date. Detailed operational definitions for specific infection sites—including pulmonary, bloodstream, and other distinct clinical syndromes—are systematically outlined in Table 1. This structured approach facilitates a rigorous distinction between active infection and colonization without enumerating standard diagnostic criteria within the text.

**Table 1:** Operational definitions and time windows for key study variables.

Variable domain	Variable	Operational definition	Time window or reference point
Time anchor	Index date	Collection date of the first eligible *S. maltophilia*-positive clinical specimen	During index hospitalization
Cohort eligibility	Active infection	Positive culture plus compatible clinical evidence and physician-documented infection; antimicrobial treatment alone was not sufficient for classification	At or around index date
Cohort eligibility	Colonization or contamination	Positive culture without compatible clinical, radiological, inflammatory, or treatment evidence of infection	At or around index date
Infection site	Pulmonary infection	Respiratory isolate plus clinical evidence of lower respiratory tract infection, such as respiratory symptoms, imaging abnormality, inflammatory response, oxygenation deterioration, or physician-documented pneumonia	At index date
Infection site	Bloodstream infection	Positive blood culture plus systemic signs and physician assessment supporting true bacteremia	At index date
Baseline factor	Age and sex	Extracted from electronic medical record	At admission or index hospitalization
Baseline factor	CCI	Charlson Comorbidity Index calculated from pre-existing chronic diseases	Before or at index date
Severity factor	ICU admission	ICU admission documented around infection onset	Around index date
Severity factor	Septic shock	Defined according to Sepsis-3 criteria	Around index date
Severity factor	Tracheal intubation or mechanical ventilation	Documented respiratory support around infection onset	Around index date
Device-related factor	Central venous catheterization	Central venous catheter documented around infection onset	Around index date
Device-related factor	Urinary catheterization	Urinary catheter documented around infection onset	Around index date
Nutritional support	Total parenteral nutrition	TPN documented around infection onset	Around index date
Immunosuppression	Chemotherapy, glucocorticoids, or other immunosuppressive treatment	Documented treatment before infection onset	Within 14 days before index date
Prior antimicrobial exposure	Carbapenem exposure	Use of meropenem, imipenem/cilastatin, or ertapenem	Within 14 days before index date
Prior antimicrobial exposure	Broad-spectrum antimicrobial exposure	Documented systemic use of broad-spectrum antibacterial agents	Within 14 days before index date
Early treatment	Initial empirical therapy	Systemic antimicrobial treatment given before susceptibility-guided adjustment	From specimen collection to 48 h after index date
Early treatment	Appropriate empirical therapy	At least one administered agent with *in vitro* activity against the first eligible isolate according to laboratory susceptibility interpretation	Within 48 h after index date
Laboratory indicators	Infection-onset laboratory values	Result closest to specimen collection date	Preferably within 24 h before or after index date
Outcome	In-hospital mortality	All-cause death before discharge from index hospitalization	During index hospitalization
Outcome	Time to death	Interval from index date to in-hospital death	During index hospitalization

### 2.4 Baseline characteristics and clinical severity variables

Baseline demographic information included age and sex. Age was extracted from the medical record and analyzed as a continuous variable. Chronic comorbidities were identified from diagnoses documented before or at the index date. The Charlson Comorbidity Index (CCI) was calculated from pre-existing comorbid conditions according to the original Charlson method and was used as an indicator of baseline comorbidity burden (15).

Clinical severity variables were systematically abstracted from the electronic medical record based on their presence around infection onset. Standardized frameworks were employed for critical classifications; specifically, the Sepsis-3 criteria were utilized to define septic shock (16), while the SOFA and APACHE II scores served as established severity measures for ICU-admitted patients (17, 18). Comprehensive operational definitions and strict time windows for all baseline characteristics, severity indicators, device-related factors, and immunosuppressive status are consolidated in Table 1.

### 2.5 Prior antimicrobial exposure

Prior antimicrobial exposure was strictly assessed during the 14 days preceding the index date. The specific criteria utilized to classify broad-spectrum antibacterial use and targeted carbapenem exposure are explicitly delineated in Table 1. Other exposure categories included penicillins or beta-lactam/beta-lactamase inhibitor combinations, cephalosporins, fluoroquinolones, sulfonamides, glycopeptides, antifungal agents, and combination antimicrobial therapy. Combination therapy was recorded when two or more antimicrobial agents or antimicrobial classes were documented during the 14-day pre-index period.

Antimicrobial exposure was extracted from electronic medical records and medication order information. When available, the recorded antimicrobial type and duration were used to support exposure classification. Antimicrobial use initiated after the index date was not counted as prior exposure.

### 2.6 Empirical therapy and appropriateness classification

The temporal parameters and clinical prerequisites defining both initial and appropriate empirical therapy are structurally detailed in Table 1. It should be noted that the classification for treatment appropriateness was based primarily on documented *in vitro* susceptibility profiles and did not systematically incorporate pharmacokinetic/pharmacodynamic (PK/PD) target attainment, optimal weight-based dosing, or renal function adjustments.

The judgment of activity was based on antimicrobial susceptibility results whenever available and followed the interpretive categories reported by the hospital microbiology laboratory. Antimicrobial agents assessed in the susceptibility profile included trimethoprim-sulfamethoxazole, doxycycline, ceftazidime, cefoperazone/sulbactam, levofloxacin, tigecycline, ticarcillin/clavulanate, ciprofloxacin, minocycline, and colistin. For agents without *S. maltophilia*-specific clinical breakpoints or with locally defined interpretive rules, the laboratory-reported result was summarized as *in vitro* activity and was not treated as direct evidence of clinical efficacy. Empirical therapy was classified as inappropriate when no agent interpreted as susceptible or clinically active against the isolate was administered within 48 h after specimen collection.

When the empirical regimen included more than one antimicrobial agent, the regimen was considered appropriate if at least one administered agent had documented *in vitro* activity against the isolate. Empirical therapy was evaluated as an exposure variable and was not treated as proof of causality, because treatment decisions in retrospective data may be influenced by baseline severity, physician judgment, local prescribing practice, and the availability and timing of culture and susceptibility reports.

### 2.7 Laboratory variables and antimicrobial susceptibility results

Laboratory indicators were extracted using results closest to the index date. The preferred window was 24 h before to 24 h after specimen collection. If more than one result was available within this window, the value nearest to the specimen collection date was used. Laboratory variables included white blood cell count, platelet count, hemoglobin, C-reactive protein, procalcitonin, aspartate aminotransferase, alanine aminotransferase, lactate dehydrogenase, albumin, and other routinely measured indicators available in the electronic medical record.

Antimicrobial susceptibility results were obtained from the microbiology laboratory records for the first eligible isolate. Species identification was systematically executed utilizing matrix-assisted laser desorption/ionization time-of-flight mass spectrometry. Concurrent antimicrobial susceptibility testing was predominantly conducted via automated platforms including the VITEK 2 system, supplemented by standard broth microdilution methods to ensure analytical accuracy. Antimicrobial susceptibility categories were rigorously interpreted in accordance with the Clinical and Laboratory Standards Institute M100 guidelines (19). Because species-specific breakpoints for *S. maltophilia* are restricted to select agents including trimethoprim-sulfamethoxazole and levofloxacin, the susceptibility thresholds for other evaluated compounds were determined by extrapolating established CLSI criteria for Enterobacterales; furthermore, United States Food and Drug Administration criteria were specifically applied for the *in vitro* assessment of tigecycline.

### 2.8 Outcome definition

The primary outcome was all-cause in-hospital mortality during the index hospitalization. Patients were classified as non-survivors if death occurred before discharge. Patients discharged alive were classified as survivors. When time-to-death information was available, it was calculated as the interval from the index date to in-hospital death. Deaths after discharge were not included because post-discharge follow-up was not part of the available dataset.

### 2.9 Data extraction, coding, and missing data

Data were extracted from electronic medical records, laboratory records, microbiology reports, medication information, and discharge records using predefined variable definitions. Extracted fields included demographic characteristics, specimen type, clinical diagnosis, department, specimen collection date, hospitalization duration, comorbidities, clinical manifestations, surgical history, infection site, ICU admission, septic shock, invasive device use, ventilation status, total parenteral nutrition, ICU severity scores where available, CCI, immunosuppressive status, prior antimicrobial exposure, antimicrobial type and duration, empirical treatment information, laboratory indicators, co-isolated microorganisms, antimicrobial susceptibility results, and in-hospital outcome.

Categorical variables were coded according to documented yes/no status or clinically interpretable categories in the medical record. Continuous variables were retained as recorded. Missing or unverifiable data were not imputed. For descriptive analyses, the available denominator was used where appropriate. For regression analyses, variables with substantial missingness or insufficient events were not forced into the multivariable model.

### 2.10 Statistical analysis

Categorical variables were summarized as numbers and percentages. Continuous variables were assessed for distribution using the Shapiro–Wilk test. Normally distributed continuous variables were reported as means with standard deviations and compared using the independent-samples *t*-test. Non-normally distributed continuous variables were reported as medians with interquartile ranges and compared using the Mann–Whitney *U*-test. Categorical variables were compared using the chi-square test or Fisher exact test, as appropriate.

Univariable logistic regression was first used to examine associations between candidate variables and in-hospital mortality. Variables with a univariable *p* value < 0.10 were considered for multivariable logistic regression, together with clinically important variables specified *a priori*, including age, sex, and CCI. Because the number of mortality events was limited, the multivariable model was restricted according to clinical relevance, event number, and avoidance of overfitting. Variables with substantial conceptual overlap, such as ICU admission, septic shock, mechanical ventilation or tracheal intubation, and invasive catheterization, were reviewed before simultaneous model entry. Multicollinearity was assessed using the variance inflation factor, with a value >5 considered indicative of relevant collinearity (20). Adjusted odds ratios (aORs) with 95% confidence intervals (CIs) were reported.

The main analysis focused on factors associated with all-cause in-hospital mortality. Particular attention was given to prior carbapenem exposure and appropriate empirical therapy because these variables were clinically relevant and central to the study question. All statistical analyses were performed using SPSS version 26.0 (IBM Corp., Armonk, NY, USA). A two-sided *p* value < 0.05 was considered statistically significant.

## 3 Results

### 3.1 Cohort structure, infection sites, and baseline clinical profile

As shown in the patient screening flow diagram in Figure 1, 670 hospitalized patients with at least one *Stenotrophomonas maltophilia*-positive clinical specimen were screened between 1 June 2023 and 31 July 2025. After exclusion of 212 records that did not meet the predefined eligibility criteria, 458 patients with confirmed *S. maltophilia* infection and available in-hospital outcome information were included in the final analytic cohort.

The cohort consisted predominantly of older hospitalized patients with substantial clinical complexity. The median age was 68 years, and 66.2% of patients were male. Pulmonary infection was the main infection site, accounting for 416 of 458 cases (90.8%), followed by urinary tract infection in 15 patients (3.3%) and other documented infection sites in 27 patients (5.9%). Because respiratory specimens constituted the dominant source of infection, the distribution of infection sites is provided in Supplementary Figure S1 to support interpretation of the infection-site structure and the infection-vs.-colonization assessment described in the Methods. ICU admission was required in 60.8% of patients, indicating that *S. maltophilia* infection was frequently identified in patients with severe illness or complex inpatient courses.

Overall, 54 patients died during the index hospitalization and 404 were discharged alive, giving an all-cause in-hospital mortality rate of 11.8% (54/458). Baseline characteristics, infection-related variables, prior antimicrobial exposure, empirical therapy classification, severity indicators, and laboratory findings were compared between non-survivors and survivors in Table 2 using variable-specific available denominators. Rather than reflecting a single isolated risk factor, the non-survivor profile showed a consistent pattern of older age, greater comorbidity burden, more intensive organ support, and a stronger inflammatory response around infection onset. Non-survivors were older and had a higher CCI, suggesting greater baseline host vulnerability. They also had higher frequencies of ICU admission, septic shock, central venous catheterization, and mechanical ventilation. Laboratory findings followed the same direction, with higher C-reactive protein and procalcitonin levels among non-survivors. Albumin tended to be lower among non-survivors, although the between-group difference did not reach statistical significance.

**Table 2:** Baseline characteristics of patients with *Stenotrophomonas maltophilia* infection stratified by in-hospital survival status.

Characteristic	Non-survivors *n* = 54	Survivors *n* = 404	*p*-value
Age, years	74.0 (60.2–81.8)	67.0 (56.0–75.0)	0.003
Male sex	35/54 (64.8)	268/404 (66.3)	0.945
Pulmonary infection	52/54 (96.3)	364/404 (90.1)	0.206
ICU admission	41/54 (75.9)	232/396 (58.6)	0.022
Septic shock	11/54 (20.4)	37/396 (9.3)	0.026
Central venous catheterization	36/51 (70.6)	166/364 (45.6)	0.001
Mechanical ventilation	27/43 (62.8)	104/271 (38.4)	0.004
Total parenteral nutrition	3/51 (5.9)	16/364 (4.4)	0.906
Chemotherapy or immunosuppression	7/51 (13.7)	30/353 (8.5)	0.349
Carbapenem exposure	48/54 (88.9)	353/395 (89.4)	0.915
Broad-spectrum antibiotic exposure	25/54 (46.3)	114/396 (28.8)	0.014
Appropriate empirical therapy	35/54 (64.8)	244/396 (61.6)	0.760
SOFA score	2.5 (0.0–10.2)	0.0 (0.0–5.0)	0.001
Charlson comorbidity index	3.0 (2.0–6.0)	2.0 (1.0–4.0)	0.002
C-reactive protein, mg/L	60.1 (15.1–141.9)	34.6 (7.6–98.0)	0.021
White blood cell count, × 10^9^/L	11.4 (7.6–17.1)	9.3 (6.2–13.4)	0.068
Albumin, g/L	33.0 (29.3–37.4)	35.2 (30.5–39.1)	0.095
Procalcitonin, ng/mL	0.9 (0.2–3.6)	0.5 (0.1–1.7)	0.013

Data are presented as median (interquartile range) or n/N (%). The survivor group included 404 patients discharged alive; denominators in the table reflect variable-specific available data because some covariate documentation was missing or not verifiable. For variables with a 396 survivor denominator, eight survivor records were unavailable for that specific covariate extraction. ICU, intensive care unit; SOFA, Sequential Organ Failure Assessment.

Prior carbapenem exposure was common in both groups and did not differ significantly between non-survivors and survivors. Appropriate empirical therapy was also comparable between groups. Broad-spectrum antibiotic exposure was more frequent among non-survivors in the descriptive baseline comparison based on available data; however, this finding was interpreted cautiously because prior antimicrobial use may reflect preceding illness complexity rather than a direct mortality determinant. To provide a clearer visual summary of these descriptive between-group patterns, selected host-vulnerability, severity-related, organ-support, and treatment-related variables from Table 2 are summarized in Supplementary Figure S2. This supplementary profile highlights that non-survivors showed greater baseline vulnerability and illness severity, whereas prior carbapenem exposure and appropriate empirical therapy were broadly comparable between survival groups.

### 3.2 Antimicrobial susceptibility profile

**Figure 2:**
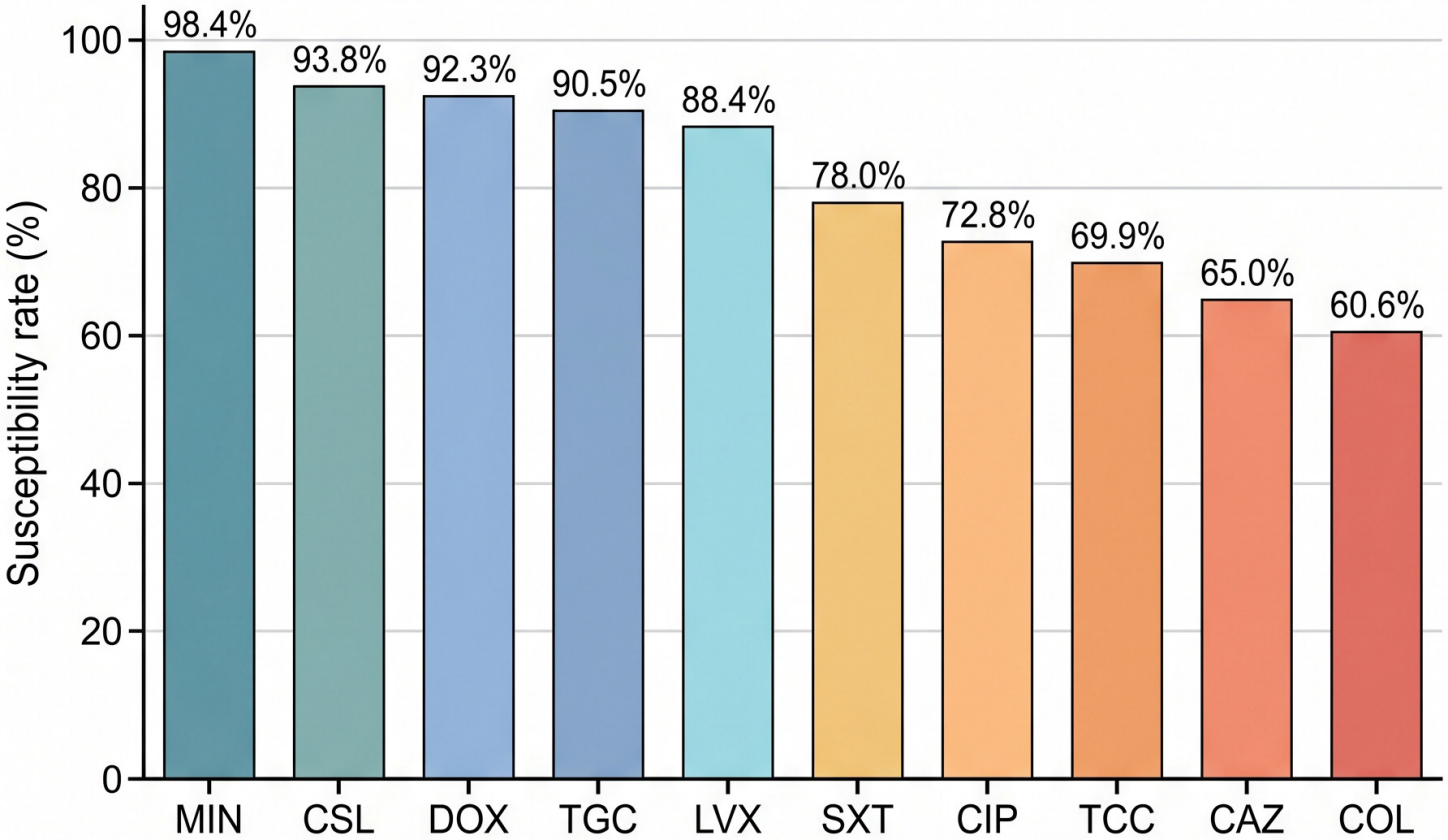
*In vitro* antimicrobial susceptibility profile of *Stenotrophomonas maltophilia* isolates. Susceptibility rates of the first eligible *S. maltophilia* isolate from each patient are detailed for the tested antimicrobial agents and are strictly ordered from highest to lowest. MIN, minocycline; CSL, cefoperazone/sulbactam; DOX, doxycycline; TGC, tigecycline; LVX, levofloxacin; SXT, trimethoprim-sulfamethoxazole; CIP, ciprofloxacin; TCC, ticarcillin/clavulanate; CAZ, ceftazidime; COL, colistin.

The antimicrobial susceptibility profile of the first eligible *S. maltophilia* isolate from each patient is presented in Figure 2. This figure summarizes the microbiological treatment context of the cohort rather than defining mortality risk directly. The highest *in vitro* susceptibility rates were observed for minocycline (98.4%), cefoperazone/sulbactam (93.8%), doxycycline (92.3%), tigecycline (90.5%), and levofloxacin (88.4%). Trimethoprim-sulfamethoxazole remained active against 78.0% of tested isolates, whereas colistin showed the lowest susceptibility rate among the tested agents, at 60.6%.

This susceptibility pattern indicates that several non-carbapenem agents retained substantial *in vitro* activity against *S. maltophilia* in this cohort. At the same time, susceptibility results should be interpreted together with host status, illness severity, drug exposure at the infection site, and the interpretive criteria used by the microbiology laboratory. Because validated *S. maltophilia*-specific clinical breakpoints are not uniformly available for all tested agents, *in vitro* activity alone may not fully explain clinical outcomes in severely ill patients.

### 3.3 Univariable analysis of factors associated with in-hospital mortality

Univariable logistic regression was performed to identify candidate variables associated with in-hospital mortality before multivariable adjustment. The results are summarized in Table 3. Several variables reflecting host vulnerability and clinical severity were associated with increased mortality, including higher CCI, older age, mechanical ventilation, central venous catheterization, urinary catheterization, septic shock, prior broad-spectrum antibiotic exposure, higher white blood cell count, and ICU admission. Lower hemoglobin was also associated with increased mortality, whereas C-reactive protein and platelet count showed borderline associations. Procalcitonin was higher among non-survivors in the descriptive comparison but was not significant when modeled as a linear continuous variable in univariable logistic regression.

**Table 3:** Univariable logistic regression analysis of factors associated with in-hospital mortality.

Variable	Odds ratio (95% CI)	*p*-value
Charlson comorbidity index, per 1-point increase	1.231 (1.106–1.371)	< 0.001
Mechanical ventilation	2.835 (1.589–5.057)	< 0.001
Central venous catheterization	2.788 (1.530–5.080)	< 0.001
Hemoglobin, per 1 g/L increase	0.983 (0.973–0.994)	0.002
Age, per 1-year increase	1.033 (1.009–1.057)	0.006
Urinary catheterization	3.408 (1.420–8.180)	0.006
Septic shock	2.475 (1.177–5.207)	0.017
White blood cell count, per 1 × 10^9^/L increase	1.027 (1.004–1.052)	0.022
ICU admission	2.028 (1.069–3.850)	0.030
C-reactive protein, per 1 mg/L increase	1.003 (1.000–1.007)	0.073
Platelet count, per 1 × 10^9^/L increase	0.997 (0.994–1.000)	0.079
Albumin, per 1 g/L increase	0.961 (0.916–1.009)	0.107
Chemotherapy or immunosuppression	1.704 (0.669–4.337)	0.264
Total parenteral nutrition	1.393 (0.392–4.948)	0.608
Procalcitonin, per 1 ng/mL increase	1.002 (0.989–1.014)	0.798
Male sex	0.935 (0.515–1.697)	0.825
Broad-spectrum antibiotic exposure	2.132 (1.197–3.799)	0.010
Appropriate empirical therapy	1.148 (0.634–2.079)	0.650
Carbapenem exposure	0.952 (0.384–2.358)	0.915

OR, odds ratio; CI, confidence interval; ICU, intensive care unit. For binary variables, the OR compares the exposed or present group with the unexposed or absent group. Procalcitonin was modeled as a linear continuous variable; because of skewness, this estimate should be interpreted together with the non-parametric descriptive comparison in Table 2.

These findings suggest that mortality was not concentrated around antimicrobial exposure alone. Instead, the univariable results showed a broader clinical pattern in which baseline comorbidity burden, organ support, invasive procedures, systemic inflammatory response, and prior broad-spectrum antibiotic exposure were related to death before adjustment. In contrast, prior carbapenem exposure was not associated with mortality. Appropriate empirical therapy also did not show a significant univariable association with survival, indicating that treatment-related variables should be interpreted in the context of baseline severity and host condition.

### 3.4 Multivariable analysis of mortality-associated factors

**Figure 3:**
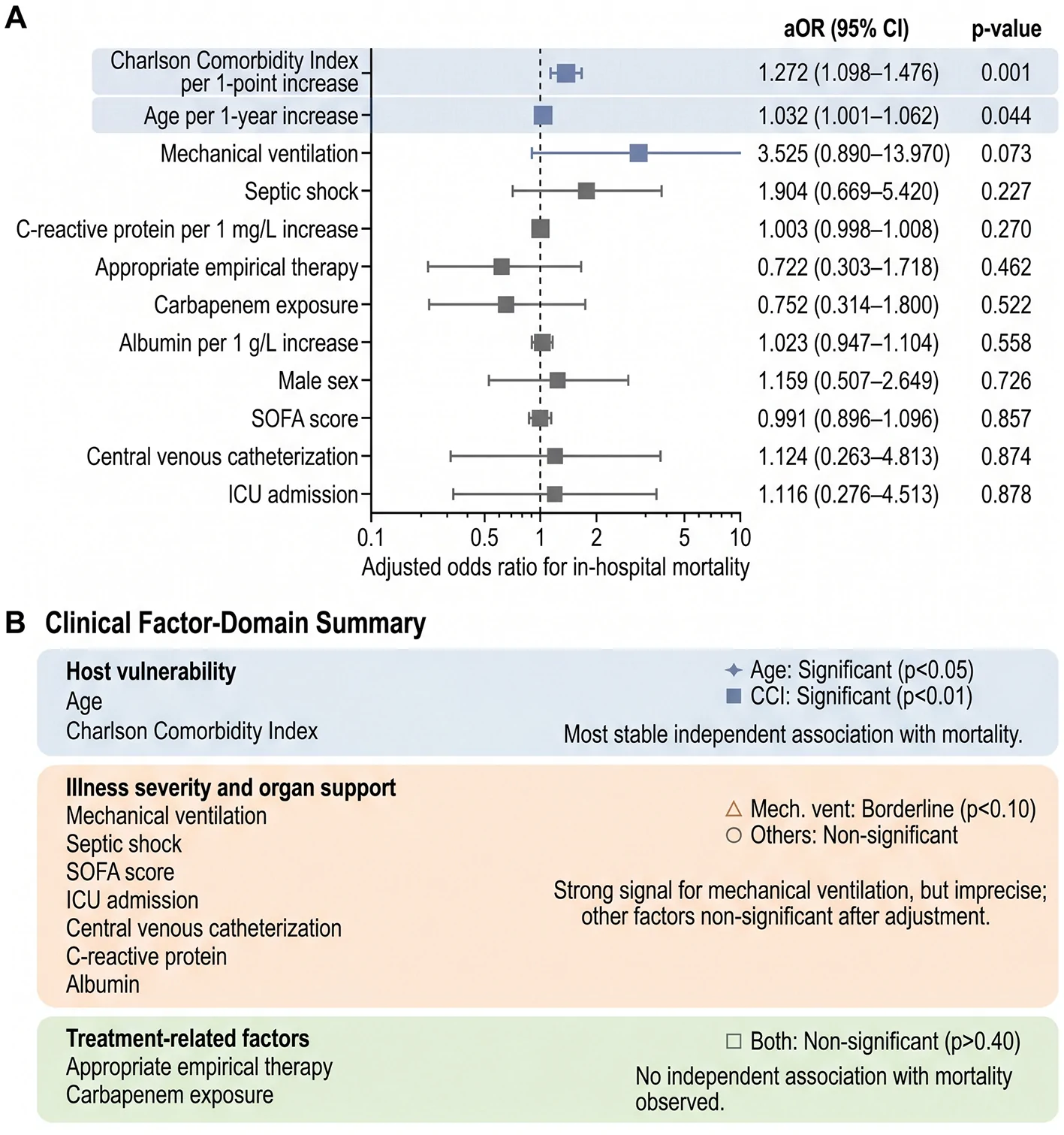
Adjusted mortality-associated factors and exploratory clinical interpretation of the multivariable model. **(A)** Forest plot showing adjusted odds ratios and 95% confidence intervals for in-hospital mortality in the exploratory multivariable logistic regression model. The vertical reference line indicates an adjusted odds ratio of 1. **(B)** Model variables are grouped into host vulnerability, illness severity and organ-support indicators, and treatment-related factors to show the clinical structure of the adjusted analysis. aOR, adjusted odds ratio; CI, confidence interval; CCI, Charlson Comorbidity Index; SOFA, Sequential Organ Failure Assessment; ICU, intensive care unit.

Variables were selected for exploratory multivariable logistic regression according to clinical relevance, univariable screening, event number, variable-specific missingness, and avoidance of model overfitting. Specifically, candidate variables including hemoglobin, white blood cell count, platelet count, and urinary catheterization met the univariable significance threshold but were ultimately excluded from the final multivariable model. This exclusion was necessitated by substantial missing data across these parameters, thereby ensuring the mathematical stability and reliability of the complete-case analysis. The adjusted model is presented in Table 4, and the corresponding adjusted odds ratios are visualized in Figure 3 to make the direction and precision of the associations easier to interpret. Because several severity-related variables were conceptually overlapping and only 54 deaths occurred, adjusted estimates were interpreted as explanatory rather than as a prediction model.

**Table 4:** Exploratory multivariable logistic regression analysis of factors associated with in-hospital mortality.

Variable	Adjusted OR	95% CI	*p*-value
Charlson Comorbidity Index, per 1-point increase	1.272	1.098–1.476	0.001
Age, per 1-year increase	1.032	1.001–1.062	0.044
Mechanical ventilation	3.525	0.890–13.970	0.073
Septic shock	1.904	0.669–5.420	0.227
C-reactive protein, per 1 mg/L increase	1.003	0.998–1.008	0.270
Appropriate empirical therapy	0.722	0.303–1.718	0.462
Carbapenem exposure	0.752	0.314–1.800	0.522
Albumin, per 1 g/L increase	1.023	0.947–1.104	0.558
Male sex	1.159	0.507–2.649	0.726
SOFA score	0.991	0.896–1.096	0.857
Central venous catheterization	1.124	0.263–4.813	0.874
ICU admission	1.116	0.276–4.513	0.878

Adjusted OR, adjusted odds ratio; CI, confidence interval; SOFA, Sequential Organ Failure Assessment; ICU, intensive care unit. The model used complete-case data for included variables. Given the limited number of deaths and overlap among severity indicators, estimates should be interpreted as exploratory and explanatory rather than as a definitive prediction model.

After adjustment, older age and higher CCI remained independently associated with in-hospital mortality. Each 1-year increase in age was associated with a 3.2% increase in the odds of death, and each 1-point increase in CCI was associated with a 27.2% increase in the odds of death. Mechanical ventilation showed a positive association with mortality, but the confidence interval crossed unity and the association did not reach statistical significance.

By contrast, prior carbapenem exposure and appropriate empirical therapy were not independently associated with in-hospital mortality after adjustment. This adjusted pattern supports the interpretation that mortality in this cohort was more strongly related to host vulnerability and severity-related factors than to prior carbapenem exposure or empirical treatment appropriateness alone, while recognizing the limited precision of adjusted estimates.

### 3.5 Prior antimicrobial exposure and mortality

Prior carbapenem exposure was documented in most patients, reflecting the high burden of preceding broad-spectrum antimicrobial use in this hospitalized cohort. Mortality was comparable between patients with and without prior carbapenem exposure, and carbapenem exposure was not associated with mortality in either univariable or multivariable analysis. Because approximately 89% of patients had prior carbapenem exposure and the unexposed group was small, this result should be interpreted as no independent association observed in this dataset rather than as evidence that carbapenem exposure has no clinical relevance. After accounting for age, comorbidity burden, and severity-related variables, prior carbapenem exposure did not function as an independent mortality-associated factor.

Broad-spectrum antibiotic exposure was more frequent among non-survivors in the descriptive baseline comparison and in univariable analysis. This pattern should be interpreted as a potential marker of preceding disease complexity, longer treatment courses, or more severe clinical trajectories before *S. maltophilia* isolation, rather than as direct evidence of a causal effect on mortality.

### 3.6 Empirical therapy appropriateness and mortality

Initial empirical therapy was evaluable in 450 patients and was classified as appropriate in 279 patients (62.0%). Mortality was comparable between patients who received appropriate empirical therapy and those who did not. In the multivariable model, appropriate empirical therapy showed a non-significant protective direction, but the confidence interval was wide and crossed 1. This finding should therefore be interpreted cautiously and not as evidence that empirical therapy is unimportant. Instead, it suggests that the measurable survival effect of empirical appropriateness may be difficult to separate from baseline host vulnerability and disease severity in a retrospective cohort.

## 4 Discussion

In this single-center retrospective cohort of 458 patients with confirmed *Stenotrophomonas maltophilia* infection, all-cause in-hospital mortality was associated mainly with baseline host vulnerability and clinical severity. Older age and a higher CCI remained independently associated with mortality after multivariable adjustment, whereas mechanical ventilation showed a strong positive but statistically non-significant association. In contrast, prior carbapenem exposure and the appropriateness of initial empirical antimicrobial therapy were not independently associated with in-hospital death. These findings suggest that prognosis in this cohort was shaped more by patient-level physiological reserve and illness severity than by antimicrobial exposure history or early treatment classification alone.

### 4.1 Host vulnerability and illness severity as the dominant prognostic pattern

The finding that older age and CCI were independently associated with mortality is consistent with previous observations that outcomes in *S. maltophilia* infection are strongly influenced by host condition, comorbidity burden, ICU-level illness, pneumonia or bloodstream infection, organ-support requirements, and inflammatory severity (21–29). *S. maltophilia* is often encountered in patients with prolonged hospitalization, invasive procedures, immunosuppression, or critical illness. In such patients, the organism may not behave as a high-virulence pathogen in isolation, but its clinical impact can become substantial when host defenses and physiological reserve are already impaired.

The CCI was one of the most stable predictors in the adjusted model. This is clinically plausible because comorbidity burden captures chronic vulnerability that may not be fully reflected by acute inflammatory markers or isolated severity indicators. The CCI was originally developed as a structured measure of comorbidity-related mortality risk (15), and in the present cohort it appeared to retain prognostic value in the setting of opportunistic Gram-negative infection. This supports the interpretation that mortality after *S. maltophilia* infection should not be attributed only to microbiological resistance or treatment choice, but should also be understood in relation to the patient's pre-existing disease burden.

Mechanical ventilation showed a strong positive association with mortality, although it did not reach statistical significance in the multivariable model. This result should not be interpreted as evidence of no clinical relevance. Rather, mechanical ventilation likely represented a composite marker of respiratory failure, critical illness, and organ-support intensity. Its wide confidence interval may reflect the limited number of mortality events and overlap with other severity-related variables, such as ICU admission, septic shock, and SOFA score. Therefore, the overall pattern still points to illness severity as an important component of mortality risk.

### 4.2 Prior carbapenem exposure and interpretation of antimicrobial risk

Prior carbapenem exposure was highly prevalent in this cohort, but it was not associated with mortality in either univariable or multivariable analysis. Previous studies have reported that cumulative carbapenem exposure may be associated with subsequent *S. maltophilia* infection in selected immunocompromised populations, whereas hospital outbreak investigations emphasize that transmission dynamics and infection-control context can also shape observed disease patterns (30, 31). Carbapenem use may contribute to selection pressure and microbial ecological shifts, but in a retrospective cohort of infected patients, prior carbapenem exposure may also reflect a more complex clinical trajectory before *S. maltophilia* isolation.

The present findings support a cautious interpretation. The absence of an observed independent mortality association does not mean that carbapenem exposure is clinically irrelevant, nor does it weaken the importance of antimicrobial stewardship. Instead, it suggests that prior carbapenem use may function more as a marker of preceding illness complexity than as a direct determinant of death once infection has occurred. This distinction matters clinically. Reducing unnecessary carbapenem use remains important for limiting antimicrobial resistance and preserving treatment options, but the prognosis of patients with *S. maltophilia* infection should not be inferred from carbapenem exposure alone. Risk assessment should integrate age, comorbidity burden, organ support, and acute severity indicators.

### 4.3 Empirical therapy appropriateness and mortality

Appropriate initial empirical therapy was not independently associated with in-hospital mortality in the adjusted model. This finding requires careful interpretation and should not be read as evidence that active early therapy is unimportant. In retrospective data, empirical treatment is strongly influenced by the physician's assessment of severity, local antimicrobial practice, prior antibiotic exposure, specimen source, and the time needed for microbiological confirmation. Patients who receive broader or apparently more appropriate early therapy may also be those who are clinically more severe at baseline. Comparative studies and meta-analyses evaluating trimethoprim-sulfamethoxazole, fluoroquinolones, and other available treatments have produced heterogeneous results, underscoring the difficulty of separating treatment effect from patient severity and indication bias (32–35).

The non-significant protective direction observed for appropriate empirical therapy in the multivariable model suggests that a potential benefit cannot be excluded. However, the wide confidence interval indicates limited precision. In addition, the outcome in this cohort was all-cause in-hospital mortality, which may be driven by advanced age, comorbidities, respiratory failure, malignancy, or other competing clinical conditions. These factors may attenuate the measurable effect of empirical antimicrobial appropriateness, especially when the pathogen emerges late in a complex hospitalization. Therefore, the result should be interpreted as showing that empirical therapy appropriateness did not independently explain mortality in this dataset, rather than as showing that empirical therapy has no clinical value.

### 4.4 Antimicrobial susceptibility profile and treatment implications

The antimicrobial susceptibility profile provides important microbiological context for interpreting the treatment findings. Current reviews and treatment guidance emphasize that candidate active agents for *S. maltophilia* may include trimethoprim-sulfamethoxazole, levofloxacin, minocycline, cefiderocol, and combination strategies in selected severe infections, but the evidence base remains largely observational and context-dependent (36–38). Minocycline, cefoperazone/sulbactam, doxycycline, tigecycline, and levofloxacin showed relatively high *in vitro* activity, whereas trimethoprim-sulfamethoxazole remained active against a lower proportion of isolates than several alternative agents. This pattern suggests that local susceptibility data are important when selecting treatment for *S. maltophilia* infection, particularly in hospitals where resistance profiles may differ from traditional assumptions.

At the same time, *in vitro* susceptibility should not be interpreted as a direct substitute for clinical effectiveness. Drug exposure at the infection site, patient severity, organ dysfunction, immune status, source control, treatment timing, and the availability of validated interpretive breakpoints may all influence outcomes (19). This is particularly relevant in a cohort dominated by pulmonary infection and ICU-level illness. The susceptibility results therefore support the need for local microbiological surveillance and individualized antimicrobial selection, but they do not by themselves explain mortality risk.

### 4.5 Clinical implications

The findings have several practical implications. First, clinicians should recognize that patients with *S. maltophilia* infection are heterogeneous and that mortality risk is not determined by the organism alone. Older patients and those with higher comorbidity burden may require earlier risk stratification and closer monitoring. Second, carbapenem exposure should be interpreted in context. It may signal prior broad-spectrum treatment and clinical complexity, but it should not be used as an isolated prognostic marker. Third, appropriate empirical therapy remains clinically important, but its survival impact may be difficult to demonstrate in retrospective cohorts where host vulnerability and critical illness dominate the clinical course. Finally, local susceptibility patterns should be incorporated into treatment decisions, especially when susceptibility to traditional first-line agents is not uniformly high. These implications are consistent with antimicrobial stewardship principles, global prioritization of antimicrobial-resistant pathogens, and surveillance data showing the ongoing clinical importance of multidrug-resistant non-fermenting Gram-negative organisms (39–41).

### 4.6 Limitations

This study has several limitations. First, it was a single-center retrospective study, and the findings may not be generalizable to hospitals with different patient populations, antimicrobial practices, or microbiological profiles. Second, although predefined criteria were used to distinguish infection from colonization, misclassification remains possible, especially for respiratory isolates. This is particularly relevant because pulmonary infection accounted for most cases in the cohort. Third, antimicrobial exposure and empirical therapy were assessed from retrospective medical records, and treatment decisions may have been influenced by unmeasured clinical factors and confounding by indication. Furthermore, prior carbapenem and broad-spectrum antimicrobial exposures were evaluated as binary variables rather than continuous days of therapy. This methodological constraint precludes the differentiation between transient perioperative prophylaxis and prolonged therapeutic courses, thereby limiting a more granular assessment of cumulative antimicrobial selection pressure. Fourth, the number of mortality events was limited relative to the number of candidate predictors, which may have reduced the precision of some adjusted estimates and increased the risk of overfitting. Fifth, the high prevalence of carbapenem exposure reduced the size of the unexposed comparison group and limited the power to detect modest exposure-related differences. Sixth, the primary outcome was all-cause in-hospital mortality rather than infection-attributable mortality or 30-day mortality; therefore, deaths related to underlying disease progression may have contributed to the observed outcome. Seventh, variable-specific missingness and the availability of ICU severity scores may have affected some subgroup and regression analyses. Finally, the study did not evaluate detailed pharmacokinetic exposure (such as PK/PD target attainment, optimal weight-based dosing, and specific renal function adjustments), adequacy and timeliness of source control, treatment duration, susceptibility breakpoint standards for all tested agents, or post-discharge outcomes. The lack of systematic adjustment for source control interventions—such as the prompt removal of infected central venous catheters or the adequate drainage of intra-abdominal abscesses—acts as a significant unmeasured confounder. Consequently, the potential presence of unresolved infectious foci alongside sub-therapeutic dosing in this retrospective cohort could have masked the true clinical survival benefit of early active empirical therapy.

## 5 Conclusions

In this retrospective cohort of 458 patients with confirmed *Stenotrophomonas maltophilia* infection, older age and higher comorbidity burden were independently associated with all-cause in-hospital mortality. Prior carbapenem exposure and appropriate empirical therapy were not independently associated with death after adjustment for host-related and severity-related factors, although these findings should be interpreted in light of the high prevalence of carbapenem exposure, variable-specific missingness, and the all-cause in-hospital mortality endpoint. These findings suggest that prognosis in *S. maltophilia* infection is driven largely by baseline vulnerability and illness severity. Clinical management should therefore combine timely antimicrobial selection based on local susceptibility patterns with early identification of high-risk patients, careful assessment of comorbidity burden, and intensified supportive care when needed.

## Data Availability

The raw data supporting the conclusions of this article will be made available by the authors, without undue reservation.

## References

[1] Brooke JS. *Stenotrophomonas maltophilia*: an emerging global opportunistic pathogen. Clin Microbiol Rev. (2012) 25:2–41. doi: 10.1128/CMR.00019-11 22232370 PMC3255966

[2] Looney WJ Narita M Muhlemann K. *Stenotrophomonas maltophilia*: an emerging opportunist human pathogen. Lancet Infect Dis. (2009) 9:312–23. doi: 10.1016/S1473-3099(09)70083-0 19393961

[3] Denton M Kerr KG. Microbiological and clinical aspects of infection associated with *Stenotrophomonas maltophilia*. *Clin Microbiol Rev*. (1998) 11:57–80. doi: 10.1128/CMR.11.1.57 PMC121376 9457429

[4] Brooke JS. Advances in the microbiology of *Stenotrophomonas maltophilia*. *Clin Microbiol Rev*. (2021) 34:e00030-19. doi: 10.1128/CMR.00030-19 34043457 PMC8262804

[5] Ryan RP Monchy S Cardinale M Taghavi S Crossman L Avison MB . The versatility and adaptation of bacteria from the genus Stenotrophomonas. Nat Rev Microbiol. (2009) 7:514–25. doi: 10.1038/nrmicro2163 19528958

[6] Adegoke AA Stenstrom TA Okoh AI. *Stenotrophomonas maltophilia* as an emerging ubiquitous pathogen: looking beyond contemporary antibiotic therapy. Front Microbiol. (2017) 8:2276. doi: 10.3389/fmicb.2017.02276 29250041 PMC5714879

[7] Chang YT Lin CY Chen YH Hsueh PR. Update on infections caused by *Stenotrophomonas maltophilia* with particular attention to resistance mechanisms and therapeutic options. Front Microbiol. (2015) 6:893. doi: 10.3389/fmicb.2015.00893 26388847 PMC4557615

[8] Sanchez MB. Antibiotic resistance in the opportunistic pathogen *Stenotrophomonas maltophilia*. *Front Microbiol*. (2015) 6:658. doi: 10.3389/fmicb.2015.00658 26175724 PMC4485184

[9] Crossman LC Gould VC Dow JM Vernikos GS Okazaki A Sebaihia M . The complete genome, comparative and functional analysis of *Stenotrophomonas maltophilia* reveals an organism heavily shielded by drug resistance determinants. Genome Biol. (2008) 9:R74. doi: 10.1186/gb-2008-9-4-r74 18419807 PMC2643945

[10] Yamada K Ishii Y Tateda K. Diversity of blaL1-like genes in *Stenotrophomonas* species: insights from genome analysis of publicly available genome sequences. Antimicrob Agents Chemother. (2023) 67:e0067323. doi: 10.1128/aac.00673-23 37584548 PMC10508171

[11] Pompilio A Crocetta V Confalone P Nicoletti M Petrucca A Guarnieri S . Adhesion to and biofilm formation on IB3-1 bronchial cells by *Stenotrophomonas maltophilia* isolates from cystic fibrosis patients. BMC Microbiol. (2010) 10:102. doi: 10.1186/1471-2180-10-102 20374629 PMC2858031

[12] Bhaumik R Aungkur NZ Anderson GG. A guide to *Stenotrophomonas maltophilia* virulence capabilities, as we currently understand them. Front Cell Infect Microbiol. (2024) 13:1322853. doi: 10.3389/fcimb.2023.1322853 38274738 PMC10808757

[13] Groschel MI Meehan CJ Barilar I Diricks M Gonzaga A Steglich M . The phylogenetic landscape and nosocomial spread of the multidrug-resistant opportunist *Stenotrophomonas maltophilia*. *Nat Commun*. (2020) 11:2044. doi: 10.1038/s41467-020-15123-0 PMC7184733 32341346

[14] Horan TC Andrus M Dudeck MA. CDC/NHSN surveillance definition of health care-associated infection and criteria for specific types of infections in the acute care setting. Am J Infect Control. (2008) 36:309–32. doi: 10.1016/j.ajic.2008.03.002 18538699

[15] Charlson ME Pompei P Ales KL MacKenzie CR. A new method of classifying prognostic comorbidity in longitudinal studies: development and validation. J Chronic Dis. (1987) 40:373–83. doi: 10.1016/0021-9681(87)90171-8 3558716

[16] Singer M Deutschman CS Seymour CW Shankar-Hari M Annane D Bauer M . The third international consensus definitions for sepsis and septic shock (Sepsis-3). JAMA. (2016) 315:801–10. doi: 10.1001/jama.2016.0287 26903338 PMC4968574

[17] Vincent JL Moreno R Takala J Willatts S De Mendonca A Bruining H . The SOFA (sepsis-related organ failure assessment) score to describe organ dysfunction/failure. Intensive Care Med. (1996) 22:707–10. doi: 10.1007/BF01709751 8844239

[18] Knaus WA Draper EA Wagner DP Zimmerman JE. APACHE II. a severity of disease classification system. Crit Care Med. (1985) 13:818–29. doi: 10.1097/00003246-198510000-00009 3928249

[19] Clinical and Laboratory Standards Institute. Performance Standards for Antimicrobial Susceptibility Testing, 35th Edn. CLSI supplement M100. Wayne, PA: Clinical and Laboratory Standards Institute (2025).

[20] Kim JH. Multicollinearity and misleading statistical results. Korean J Anesthesiol. (2019) 72:558–69. doi: 10.4097/kja.19087 31304696 PMC6900425

[21] Huang C Lin L Kuo S. Risk factors for mortality in *Stenotrophomonas maltophilia* bacteremia: a meta-analysis. Infect Dis. (2024) 56:335–47. doi: 10.1080/23744235.2024.2324365 38436567

[22] Garcia Paez JI Costa SF. Risk factors associated with mortality of infections caused by *Stenotrophomonas maltophilia*: a systematic review. J Hosp Infect. (2008) 70:101–8. doi: 10.1016/j.jhin.2008.05.020 18621440

[23] Kim EJ Kim YC Ahn JY Jeong SJ Ku NS Choi JY . Risk factors for mortality in patients with *Stenotrophomonas maltophilia* bacteremia and clinical impact of quinolone-resistant strains. BMC Infect Dis. (2019) 19:754. doi: 10.1186/s12879-019-4394-4 31462215 PMC6714101

[24] Lee YH Lee J Yu B Lee WK Choi SH Park JE . Risk factors for mortality in intensive care unit patients with *Stenotrophomonas maltophilia* pneumonia in South Korea. Acute Crit Care. (2023) 38:442–51. doi: 10.4266/acc.2023.00682 37994018 PMC10718495

[25] Gezer Y Taysi MR Tarakci A Gokce O Danaci G . Evaluation of clinical outcomes and risk factors associated with mortality in patients with *Stenotrophomonas maltophilia* bloodstream infection: a multicenter study. BMC Infect Dis. (2024) 24:1387. doi: 10.1186/s12879-024-10293-4 39639202 PMC11619100

[26] Ma R Chen Q Huang Y Cheng Z Wang X Xia L . The prognosis of patients tested positive for *Stenotrophomonas maltophilia* from different sources. Infect Drug Resist. (2023) 16:4779–87. doi: 10.2147/IDR.S417151 37520451 PMC10377593

[27] Kwa AL Low JG Lim TP Leow PC Kurup A Tam VH. Independent predictors for mortality in patients with positive *Stenotrophomonas maltophilia* cultures. Ann Acad Med Singap. (2008) 37:826–30. doi: 10.47102/annals-acadmedsg.V37N10p826 19037515

[28] Kanchanasuwan S Rongmuang J Siripaitoon P Kositpantawong N Charoenmak B Hortiwakul T . Clinical characteristics, outcomes, and risk factors for mortality in patients with *Stenotrophomonas maltophilia* bacteremia. J Clin Med. (2022) 11:3085. doi: 10.3390/jcm11113085 35683471 PMC9181236

[29] Hamdi AM Fida M Abu Saleh OM Beam E. *Stenotrophomonas* bacteremia antibiotic susceptibility and prognostic determinants: mayo clinic 10-year experience. Open Forum Infect Dis. (2020) 7:ofaa008. doi: 10.1093/ofid/ofaa008 32016126 PMC6988836

[30] Aitken SL Sahasrabhojane PV Kontoyiannis DP . Alterations of the oral microbiome and cumulative carbapenem exposure are associated with *Stenotrophomonas maltophilia* infection in patients with acute myeloid leukemia receiving chemotherapy. Clin Infect Dis. (2021) 72:1507–13. doi: 10.1093/cid/ciaa778 32544947 PMC8096257

[31] Cristina ML Sartini M Ottria G Schinca E Adriano G Innocenti L . *Stenotrophomonas maltophilia* outbreak in an ICU: investigation of possible routes of transmission and implementation of infection control measures. Pathogens. (2024) 13:369. doi: 10.3390/pathogens13050369 38787221 PMC11124162

[32] Ko JH Kang CI Cornejo-Juarez P Yeh KM Wang CH Cho SY . Fluoroquinolones versus trimethoprim-sulfamethoxazole for the treatment of *Stenotrophomonas maltophilia* infections: a systematic review and meta-analysis. Clin Microbiol Infect. (2019) 25:546–54. doi: 10.1016/j.cmi.2018.11.008 30448331

[33] Sarzynski SH Warner S Sun J Matsouaka R Dekker JP Babiker A . Trimethoprim-sulfamethoxazole versus levofloxacin for *Stenotrophomonas maltophilia* infections: a retrospective comparative effectiveness study of electronic health records from 154 US hospitals. Open Forum Infect Dis. (2022) 9:ofab644. doi: 10.1093/ofid/ofab644 35097154 PMC8794591

[34] Almangour TA Alkherb Z Alruwaite S Alsahli R Alali H Almohaizeie A . Trimethoprim-sulfamethoxazole versus levofloxacin for the treatment of *Stenotrophomonas maltophilia* infections: a multicentre cohort study. J Glob Antimicrob Resist. (2024) 38:42–8. doi: 10.1016/j.jgar.2024.05.016 38821443

[35] Maraolo AE Licciardi F Gentile I Saracino A Belati A Bavaro DF. *Stenotrophomonas maltophilia* infections: a systematic review and meta-analysis of comparative efficacy of available treatments, with critical assessment of novel therapeutic options. Antibiotics. (2023) 12:910. doi: 10.3390/antibiotics12050910 37237813 PMC10215754

[36] Mojica MF Humphries R LiPuma JJ Mathers AJ Rao GG Shelburne SA . Clinical challenges treating *Stenotrophomonas maltophilia* infections: an update. JAC Antimicrob Resist. (2022) 4:dlac040. doi: 10.1093/jacamr/dlac040 35529051 PMC9071536

[37] Tamma PD Heil EL Justo JA Mathers AJ Satlin MJ Bonomo RA. Infectious Diseases Society of America 2024 guidance on the treatment of antimicrobial-resistant Gram-negative infections. Clin Infect Dis. (2024) ciae403. doi: 10.1093/cid/ciae403 39108079

[38] Monardo R Mojica MF Ripa M Aitken SL Bonomo RA van Duin D. How do I manage a patient with *Stenotrophomonas maltophilia* infection? Clin Microbiol Infect. (2025) 31:1291–7. doi: 10.1016/j.cmi.2025.04.031 40339792

[39] Barlam TF Cosgrove SE Abbo LM MacDougall C Schuetz AN Septimus EJ . Implementing an antibiotic stewardship program: guidelines by the Infectious Diseases Society of America and the Society for Healthcare Epidemiology of America. Clin Infect Dis. (2016) 62:e51–77. doi: 10.1093/cid/ciw118 27080992 PMC5006285

[40] Tacconelli E Carrara E Savoldi A Harbarth S Mendelson M Monnet DL . Discovery, research, and development of new antibiotics: the WHO priority list of antibiotic-resistant bacteria and tuberculosis. Lancet Infect Dis. (2018) 18:318–27. doi: 10.1016/S1473-3099(17)30753-3 29276051

[41] Gales AC Jones RN Forward KR Linares J Sader HS Verhoef J. Emerging importance of multidrug-resistant *Acinetobacter* species and *Stenotrophomonas maltophilia* as pathogens in seriously ill patients: geographic patterns, epidemiological features, and trends in the SENTRY Antimicrobial Surveillance Program (1997–1999). Clin Infect Dis. (2001) 32(Suppl 2):S104–13. doi: 10.1086/320183 11320451

